# RNA Granules in Antiviral Innate Immunity: A Kaposi’s Sarcoma-Associated Herpesvirus Journey

**DOI:** 10.3389/fmicb.2021.794431

**Published:** 2022-01-05

**Authors:** Nishi R. Sharma, Zhi-Ming Zheng

**Affiliations:** ^1^Department of Molecular Medicine, School of Interdisciplinary Studies, Jamia Hamdard University, New Delhi, India; ^2^Tumor Virus RNA Biology Section, HIV Dynamics and Replication Program, Center for Cancer Research, National Cancer Institute, National Institutes of Health, Frederick, MD, United States

**Keywords:** virus infections, innate immunity, KSHV, ORF57, stress granule (SG), processing bodies (PB), PKR, ago

## Abstract

RNA granules are cytoplasmic, non-membranous ribonucleoprotein compartments that form ubiquitously and are often referred to as foci for post-transcriptional gene regulation. Recent research on RNA processing bodies (PB) and stress granules (SG) has shown wide implications of these cytoplasmic RNA granules and their components in suppression of RNA translation as host intracellular innate immunity against infecting viruses. Many RNA viruses either counteract or co-opt these RNA granules; however, many fundamental questions about DNA viruses with respect to their interaction with these two RNA granules remain elusive. Kaposi’s sarcoma-associated herpesvirus (KSHV), a tumor-causing DNA virus, exhibits two distinct phases of infection and encodes ∼90 viral gene products during the lytic phase of infection compared to only a few (∼5) during the latent phase. Thus, productive KSHV infection relies heavily on the host cell translational machinery, which often links to the formation of PB and SG. One major question is how KSHV counteracts the hostile environment of RNA granules for its productive infection. Recent studies demonstrated that KSHV copes with the translational suppression by cellular RNA granules, PB and SG, by expressing ORF57, a viral RNA-binding protein, during KSHV lytic infection. ORF57 interacts with Ago2 and GW182, two major components of PB, and prevents the scaffolding activity of GW182 at the initial stage of PB formation in the infected cells. ORF57 also interacts with protein kinase R (PKR) and PKR-activating protein (PACT) to block PKR dimerization and kinase activation, and thus inhibits eIF2α phosphorylation and SG formation. The homologous immediate-early regulatory protein ICP27 of herpes simplex virus type 1 (HSV-1), but not the EB2 protein of Epstein-Barr virus (EBV), shares this conserved inhibitory function with KSHV ORF57 on PB and SG. Through KSHV ORF57 studies, we have learned much about how a DNA virus in the infected cells is equipped to evade host antiviral immunity for its replication and productive infection. KSHV ORF57 would be an excellent viral target for development of anti-KSHV-specific therapy.

## Introduction

Representing the first line of defense for host cells against invading viral pathogens, the cellular innate immune system is equipped with germline-encoded pattern recognition receptors (PRRs) to recognize pathogen-derived and conserved pathogen-associated molecular patterns (PAMPs (Brennan and Bowie, 2010; Brubaker et al., 2015). This intricate framework, in response to recognition of antigens and non-self RNA/DNA, also facilitates mammalian cells to produce several inflammatory cytokines (IL-1β and IL-18, etc.) and type I interferons (IFNs) as a strategy of intrinsic defense against invading viral pathogens. Until now, six types of PRRs resulting in the transcription of type I IFNs in responses to the infections of RNA and DNA viruses have been identified, including toll-like receptors (TLRs), retinoic acid-inducible gene-I (RIG-I)-like receptors (RLRs), C-type lectin receptors (CLRs), absent in melanoma 2 (AIM2)-like receptors (ALRs), nucleotide-binding oligomerization domain (NOD)-like receptors (NLRs), and cytosolic DNA-sensing receptors such as cyclic GMP-AMP synthase (cGAS)-stimulator of interferon genes (STING; Thompson et al., 2011; Lee et al., 2016; Zevini et al., 2017; Wei and Lan, 2018; Hopfner and Hornung, 2020). At least 10 human TLRs (TLR1–TLR10), which are believed to function as dimers, are type I transmembrane proteins that are composed of an N-terminal leucine-rich repeat-containing ectodomain responsible for PAMP recognition, a transmembrane domain, and a cytoplasmic C-terminal TIR (Toll-interleukin-1 receptor homology) domain that activates downstream signal transduction (Bowie and O’Neill, 2000; Akira and Takeda, 2004). While some of these receptors (TLR1, TLR2, TLR4, TLR5, TLR6, and TLR10) reside on plasma membranes and recognize components of microbial cell walls and membranes such as lipoproteins and peptidoglycans, members of the TLR3 and TLR7 families are intracellular TLRs expressed in endosomes and lysosomes. In the battle with viral infections, TLR3 and TLR10 recognize double-stranded RNA (dsRNA; Lee et al., 2018; Wei and Lan, 2018), whereas TLR7, TLR8, and TLR9 make up the TLR7 family, with TLR7 and TLR8 detecting single-stranded RNA (ssRNA) and TLR9 recognizing unmethylated CpG DNA (Kawai and Akira, 2011).

**FIGURE 1:**
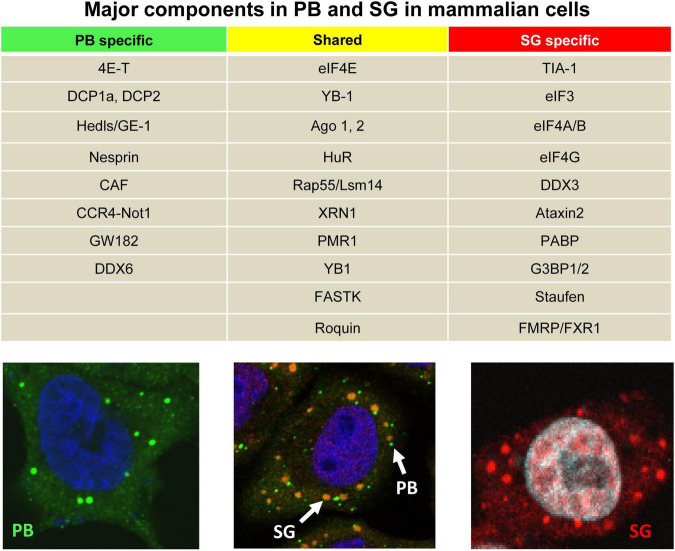
Two major cellular RNA granules and their protein components. The table shows major specific or shared cellular protein components of PB and SG in mammalian cells. In the bottom panels, PB (green) and SG (red) are visible in HeLa cells after immunostaining with an anti-GW182 antibody under physiological conditions **(left)** or with an anti-TIA-1 antibody during stress induced by treatment with 0.5 mM NaAS_2_O_3_ (arsenite or ARS) for 30 min **(right)**. Middle panel showing the HeLa cells under ARS stress were double stained with an anti-GW182antibody for PB (green) and an anti-TIA-1 antibody for SG (yellow).

The intracellular innate immune system has another dimension of antiviral defense in the form of RNA granules. In mammalian cells, formation of RNA granules allows flexibility for survival during adverse biological conditions. Two distinct non-membranous RNA granules, RNA processing bodies (P-bodies, PB) and stress granules (SG), are found in mammalian somatic cells (Figure 1), which regulate RNA metabolism and cellular homeostasis (Anderson and Kedersha, 2006, 2009). PB appear physiologically and comprise RNA and a unique composition of RNA-binding protein (RBP) markers including glycine/tryptophan-rich GW182, DDX6, and decapping/deadenylating enzymes (Eystathioy et al., 2003; Figure 1). PB are the cytoplasmic ribonucleoprotein (RNP) foci where small interfering RNA (siRNA)- or microRNA (miRNA)-guided mRNAs were once proposed the site for RNA processing and degradation (Cougot et al., 2004; Liu et al., 2005). The current observations suggest that mRNAs in PB are not degraded but remain translational after release from PB (Brengues et al., 2005; Aizer et al., 2014; Hubstenberger et al., 2017; Wilbertz et al., 2019). SG, on the other hand, appear in the cells only during stress and comprise stalled mRNAs and a unique composition of RBP markers TIA-1, G3BP1, and PABPC1 (Figure 1). SG initiate global translational arrest by storing mRNAs (Anderson and Kedersha, 2009) or exchange with either polysomes for translation or PB for RNA degradation (Anderson and Kedersha, 2009).

The formation of RNA granules is attributed to protein/RNA biomolecular condensates proposed to dynamically form by liquid-liquid phase separation, which reportedly depends on three factors: a dense network of redundant RNA-protein interactions; low-complexity sequences in proteins; and weak to intermediate interactions, including polyelectrolyte-polyelectrolyte interactions and between proteins-RNA interactions (Luo et al., 2018; Youn et al., 2019; Riggs et al., 2020). Recent reports suggest that SG and PB proteomes, in general, are biased toward the proteins with intrinsically disordered regions and have a high propensity to contain primary sequence features favoring phase separation (Protter and Parker, 2016; Protter et al., 2018). The latest success in the purification of PB using a fluorescence-activated particle sorting (FAPS)-based method from human epithelial cells helped to identify hundreds of proteins and thousands of accumulated and repressed mRNAs encoding regulatory processes (Hubstenberger et al., 2017).

A new user-friendly database^1^ collating genes or proteins associated with SG or PB has been generated (Youn et al., 2019). A similar effort to study SG (Jain et al., 2016) revealed stable substructures, referred to as cores, within SG from sodium arsenite (ARS)-stressed U2OS cells by super-resolution microscopy, which could be purified using a series of differential centrifugations and then affinity purification. Proteomic analysis of these cores revealed a dense network of protein-protein interactions linking SG with human diseases. This study proposed a model of SG assembly and dynamics requiring ATP-dependent helicases and protein-remodeling complexes as conserved SG components. The study further suggested that SG contain a stable core structure surrounded by a dynamic shell with assembly, disassembly, and transitions between the core and shell modulated by numerous protein- and RNA-remodeling complexes. Using the same SG purification protocol, this group also described the SG transcriptome of yeast and mammalian cells (Khong et al., 2017) through RNA-Seq analysis of purified SG cores and single-molecule fluorescence *in situ* hybridization (smFISH) validation. This study revealed that only ∼10% of bulk mRNA molecules are accumulated in mammalian SG and only 185 genes have more than 50% of their mRNA molecules in SG. In general, almost every mRNA, and some non-coding RNAs (ncRNAs), could be targeted to SG, although the targeting efficiency varies from <1 to >95%.

Besides its role in cellular homeostasis, formation of RNA granules also represents a new paradigm of virus-host interaction. Mammalian RNA granules can elicit an antiviral response independent of type I IFNs and both PB and SG are important components of the host cell antiviral response (Zhang et al., 2019). For instance, suppression of PB and/or SG formation enhances human immunodeficiency virus type 1 (HIV-1) and Kaposi’s sarcoma-associated herpesvirus (KSHV) production (Nathans et al., 2009; Sharma et al., 2019). A fascinating question remains, however, as to how viruses can inhibit PB and SG assembly. Moreover, virus infection imposes stress in host cells, causing a burden on the biosynthetic machineries of host cells including mRNA translation (McInerney et al., 2005) and thereby risking their own production by inducing SG. The overall understanding of whether SG are induced or suppressed by infected viruses continues to be an area of high interest. Notably, several RNA viruses show the ability to suppress the formation of SG by a viral factor (Emara and Brinton, 2007; White et al., 2007; Khaperskyy et al., 2012, 2014; Valiente-Echeverria et al., 2014). Recent reports provide profound evidence that DNA virus infections also regulate the formation of RNA granules in their host cells (Sharma et al., 2017, 2019; Zhang et al., 2019). In this brief review, we will use KSHV as an example to illustrate how a human DNA tumor virus regulates the formation of both PB and SG during KSHV lytic infection.

Kaposi’s sarcoma-associated herpesvirus, or human herpesvirus 8 (HHV8), is a γ-2 herpesvirus (Chang et al., 1994). KSHV is the etiological agent of Kaposi’s sarcoma, primary effusion lymphoma (PEL), and multicentric Castleman disease (MCD; Chang et al., 1994). Like other herpesviruses, KSHV has a large DNA genome of ∼165 kb that encodes nearly 90 open reading frames (ORFs) and several miRNAs and long non-coding RNAs (lncRNAs). Similar to other herpesviruses, KSHV, upon entering a susceptible host cell, causes lytic productive infection, but after lytic infection, establishes a latent infection within the host cell. While the latent infection features the highly restricted expression of only few viral genes (Zhong et al., 1996), the lytic program allows infectious virions to be shed and transmitted to new hosts. KSHV lytic expression commences the translation of all viral coding transcripts and thus imposes a stress on the host’s translational machinery.

## Kaposi’s Sarcoma-Associated Herpesvirus Regulation of Antiviral Processing Bodies Formation for Lytic Virus Infection and Virion Production

**FIGURE 2:**
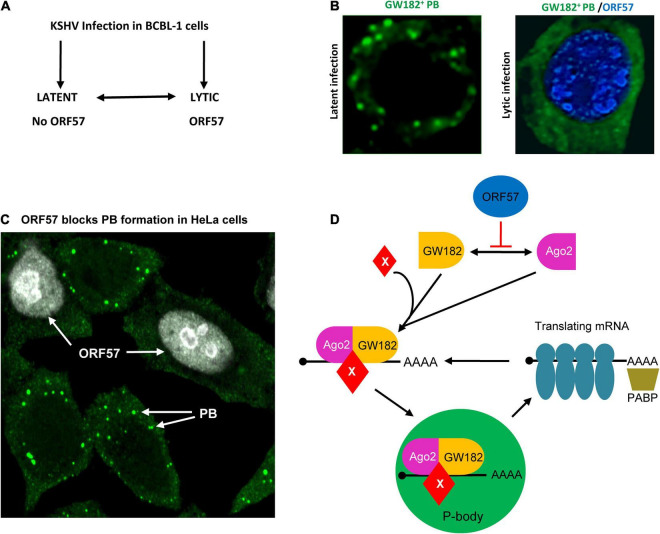
Inhibition of PB assembly during Kaposi’s sarcoma-associated herpesvirus (KSHV) lytic infection. **(A)** Flow diagram showing the exchangeable progression of lytic and latent phases of KSHV infection in BCBL-1 (B lymphocytes) cells. KSHV ORF57 is expressed only in viral lytic infection. **(B)** Left panel, a BCBL-1 cell carrying latent KSHV infection showing PB (green), after immunostaining with an anti-GW182antibody. Right panel, inhibition of PB assembly during KSHV lytic infection by expression of viral ORF57 (blue). **(C)** Inhibition of PB formation by ectopic ORF57 in HeLa cells. **(D)** Schematic model depicting the steps of KSHV ORF57 inhibition of PB formation during KSHV lytic infection by direct interaction with Ago2 and GW182. The mRNA translation can be stopped by RNA-induced silencing complex (RISC) including Ago2-GW182 complex with other factors shown as X. As a result, the translating mRNA can be stripped of ribosomes and the translational complex can be collapsed when binding to the Ago2-GW182-X complex. Panel **(D)** is modified from our previously published figure (Sharma et al., 2019).

Kaposi’s sarcoma-associated herpesvirus-infected B lymphocytes (BCBL-1, JSC-1, BC-3, and tonsil resting B cells) (Arvanitakis et al., 1996; Renne et al., 1996; Cannon et al., 2000; Hassman et al., 2011; Myoung and Ganem, 2011a) and epithelial cells (HEK293-derived Bac36 cells and the renal carcinoma cell line Caki-1-derived-iSLK.219, iSLK-Bac16 cells) (Majerciak et al., 2007; Myoung and Ganem, 2011b; Brulois et al., 2012; Sturzl et al., 2013) harbor an episomal KSHV genome at the latent stage but can be reactivated to lytic KSHV infection under hypoxia (Davis et al., 2001; Cai et al., 2006) or oxidative stress conditions (Li et al., 2011; Ye et al., 2011) or in the presence of valproic acid (Shaw et al., 2000; Majerciak et al., 2007), butyrate (Renne et al., 1996; Miller et al., 1997), or 12-*O*-Tetradecanoyl-phorbol-13-acetate (TPA; Miller et al., 1997) by inducing the expression of two viral essential genes: a viral replication and transcription activator (RTA, or ORF50) and a viral mRNA regulator and stabilizer (MTA, mRNA transcript accumulation, or ORF57) (Figure 2A).

An early observation on PB suppression during KSHV lytic infection came from a study (Corcoran et al., 2012) showing that induction of KSHV lytic gene expression in iSLK cells led to elimination of cytoplasmic PB. This study demonstrated that ectopically expressed viral G-protein-coupled receptor (vGPCR) was capable of eliminating PB, but the role of vGPCR in preventing PB formation during KSHV lytic infection in the context of the KSHV genome was not defined. However, the study claimed that overexpressed ectopic vGPCR activates RhoA subfamily GTPases, the central regulator of actin reorganization, and activates the p38/MK2 pathway for the stabilization of ARE (AU-rich elements)-containing mRNAs and inhibits PB. In a separate study, PB dispersion in endothelial cells with latent KSHV infection was shown to be affected by ectopic expression of kaposin B protein *via* activation of a non-canonical Rho-GTPase signaling axis involving MK2, hsp27, p115RhoGEF, and RhoA, but in a ROCK1/2-independent manner (Corcoran et al., 2015). As the study was not conducted in the context of the KSHV genome with physiological expression of kaposin B, the role of kaposin B in regulating PB formation during KSHV latent infection was again inconclusive.

Using anti-GW182, anti-Dcp1A, and anti-DDX6 antibodies for immunofluorescence (IF) staining assays to investigate PB formation in KSHV-infected B lymphocytes or epithelial cells, we recently revealed that BCBL-1, HEK293-derived BAC36, and Caki-1-derived iSLK-BAC16 cells with KSHV latent infection are all capable of PB formation, but when reactivated with KSHV lytic infection, they displayed no PB or a dramatical reduction of PB formation (Sharma et al., 2019; Zhang et al., 2019; Figure 2B). Suppression of PB formation during KSHV lytic infection was found to be related to viral ORF57 expression, because the BAC36 cells containing an ORF57-null KSHV genome (BAC36-Δ57) lost this inhibition on PB formation (Sharma et al., 2019; Zhang et al., 2019). Further investigation by expression of ORF57 in HeLa cells in the absence of other KSHV proteins revealed that ORF57 expression is necessary to inhibit PB formation (Figure 2C), but its N-terminal ORF57 mutant lacking the protein-protein and protein-RNA interaction activities was not (Sharma et al., 2019; Zhang et al., 2019). Interestingly, HSV-1 ICP27 but not EBV EB2, two homologs of KSHV ORF57, also showed a strong inhibitory effect on PB formation in HeLa cells (Sharma et al., 2019; Zhang et al., 2019). The reason why EBV EB2 lacks such function in suppressing PB formation remains to be understood. Surprisingly, in this study (Sharma et al., 2019; Zhang et al., 2019), we found that miRNAs and the miRNA pathway do not contribute to PB formation as previously reported (Liu et al., 2005; Eulalio et al., 2007). PB formation is independent of mature miRNAs in Dicer-knockout cells or cells with efficient knockdown expression of Dicer or Ago2, a major component of RNA-induced silencing complex (RISC). We also found that knockdown of DCP1a expression had no effect on PB formation (Sharma et al., 2019; Zhang et al., 2019).

**FIGURE 3:**
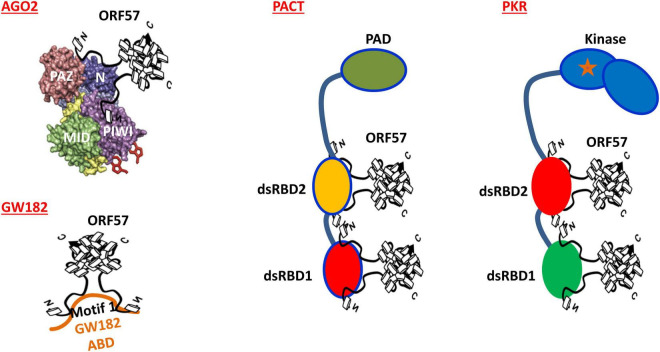
Mechanisms of how KSHV ORF57 blocks the formation of both PB and SG in mammalian cell. Dimeric ORF57 interacts with the N domain of Ago2 and the motif 1 of GW182 Ago2-binding domain (ABD) to prevent Ago2-GW182 interaction, subsequently blocking PB formation in mammalian cells. Dimeric ORF57 also directly interacts with two dsRNA-binding domains (dsRBDs) of both PACT and PKR to block PKR dimerization and PKR kinase activation, thus preventing SG formation in the cells subjected to stress.

Mechanistically, KSHV ORF57 does not affect host cells expressing Ago2 and GW182, but suppresses PB formation by direct interaction with Ago2 and GW182 and affects the scaffolding activity of GW182 at the initial stage of PB formation (Sharma et al., 2019). By co-immunoprecipitation, we found that ORF57, but not its dysfunctional mutant, interacts with the N-terminal domain of Ago2 and separately with the motif 1 of the N-terminal GW-rich, Ago2-interacting domain of GW182 (Figure 3) in an RNA-independent manner (Sharma et al., 2019) and these interactions block the Ago2-GW182 interaction (Sharma et al., 2019) required for PB formation (Yao et al., 2011; Figure 2D). Importantly, knockdown of GW182 expression in KSHV-infected iSLK-Bac16 cells was found to block PB formation, subsequently promoting production of infectious KSHV virions by ∼100-fold in the cells with reactivated lytic infection (Sharma et al., 2019). Given that PB function is an important component of host innate immunity to prevent virus infection and replication and that viral ORF57 is essential for KSHV replication and virus production (Majerciak et al., 2007; BeltCappellino et al., 2019), the identified novel function of ORF57 in blocking PB formation in the KSHV-infected host cells has greatly advanced our understanding of how a DNA tumor virus could escape the hostile surveillance of host innate immunity to benefit its infection and replication.

## Kaposi’s Sarcoma-Associated Herpesvirus Regulation of Mammalian Antiviral Stress Granules

Mammalian cells undergo a distinct stage of “translational dormancy” during stress. Here, the translating pool of mRNAs forming polysomes dissembles and the resulting mRNA-translation initiation complexes along with many RBPs, including TIA-1, G3BP1, and PABP, aggregate to form SG (Kedersha et al., 2000; Kimball et al., 2003). During cellular stress, four different kinases (general control non-derepressible 2, GCN2; protein kinase R, PKR; PKR-like endoplasmic reticulum kinase, PERK; and heme regulated inhibitory, HRI) induce phosphorylation of eIF2α, a major trigger of cascaded reactions in SG formation, depending on the cause of stress (Donnelly et al., 2013; Panas et al., 2016). Both viral infection (Lloyd, 2013; Reineke and Lloyd, 2013) and ARS (Patel and Sen, 1998) activate PKR phosphorylation, dimerization, and PKR kinase activity. ARS activates PKR kinase activity through binding with PKR-activating protein (PACT; Patel et al., 2000; Li et al., 2006). The typical SG assembly is mostly mediated by phosphorylated eIF2 (Kedersha et al., 1999), the alpha subunit of which, eIF2α, is phosphorylated by any of the four described kinases above. Thus, the formation of SG is initiated as a downstream event of elF2α phosphorylation. Normally, eIF2α is required to initiate mRNA translation by promoting the binding of tRNA^met^ to the 40S ribosome in a GTP-dependent manner. Stress induces phosphorylation of eIF2α to attenuate eIF2α activity and thereby promotes prion-like TIA-1 aggregation/nucleation to form SG where mRNA translation is stalled (Donnelly et al., 2013). Among the four well-known eIF2α kinases, PKR (the key cellular sensor of dsRNAs) also serves to induce the innate immune response through virus infection-induced production of type I IFNs which bind to an IFN-stimulated response element (ISRE) in the PKR promoter and activate PKR transcription (Kuhen and Samuel, 1997). An early report claimed that KSHV blocks PKR phosphorylation by a short, artificial form (163 aa residues) of viral IFN regulatory factor 2 (vIRF-2) (36). Unfortunately, this report was misleading, because the actual vIRF-2 ORF splits into two separate exons, with exon 1 in K11.1 and exon 2 in K11 (Jenner et al., 2001; Cunningham et al., 2003), and encodes an authentic vIRF-2 bearing 680 aa residues. In other words, the annotated vIRF-2 ORF encoding 163 aa residues in an early report (36) was wrongly predicted from the KSHV genome (Russo et al., 1996; Neipel et al., 1997). We subsequently examined the functionality of the full-length authentic vIRF-2 bearing 680 aa residues in HeLa cells with or without ARS treatment and did not find its inhibitory effect on ARS-induced SG formation (Sharma et al., 2017).

**FIGURE 4:**
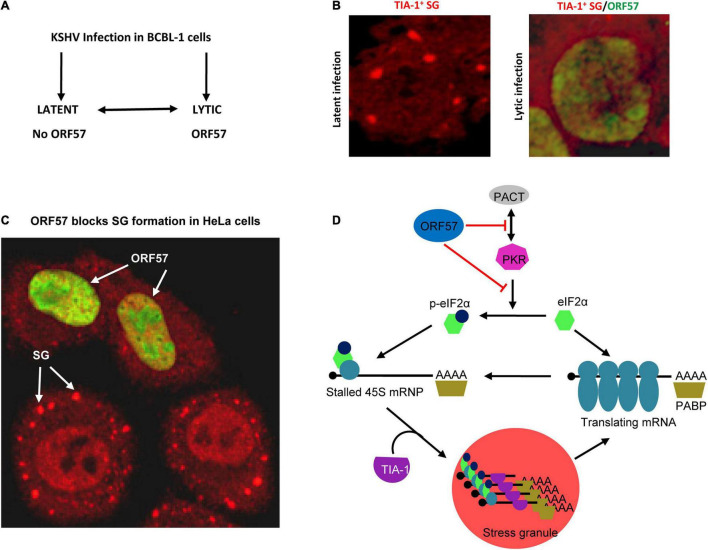
Disruption of SG assembly during KSHV lytic infection. **(A)** Flow diagram showing the exchangeable progression of lytic and latent phases of KSHV infection in BCBL-1 (B lymphocytes) cells. **(B)** Left panel, a BCBL-1 cell carrying latent KSHV infection showing SG (red) by immunostaining with an anti-TIA-1 antibody after treatment with 0.5 mM arsenite for 30 mins. Right panel, inhibition of SG assembly during KSHV lytic infection by expression of viral ORF57 (green). **(C)** Ectopic ORF57 inhibiting SG formation in HeLa cells under ARS stress. **(D)** Schematic model depicting the ORF57 interactions with PACT and PKR to block PKR dimerization and activation and thus inhibit SG formation during KSHV lytic infection. Panel **(D)** is modified from our previously published figure (Sharma et al., 2019).

Kaposi’s sarcoma-associated herpesvirus regulation of SG formation was initially observed in our recent study (Sharma et al., 2017). By using SG-specific TIA-1 antibody staining (Gilks et al., 2004), we initially did not find any TIA-1^+^ SG formation in BCBL-1 cells with latent or with lytic KSHV infection reactivated by butyrate (Figure 4A). However, after a 30-min treatment with 0.5 mM ARS, a common chemical inducer that causes oxidative stress (Kedersha et al., 1999), the BCBL-1 cells with KSHV latent infection appeared in many cytoplasmic TIA-1^+^ SG, but did not do so when the cells were reactivated with virus lytic infection (Figure 4B). Data indicate that some KSHV lytic proteins may inhibit SG formation. To explore which viral lytic proteins might contribute to this negative regulation on SG formation, we compared HEK293-derived Bac36 cells containing a wild-type (wt) KSHV genome or an ORF57-null (Δ57) KSHV genome with latent or lytic KSHV infection for SG formation under ARS treatment. We found that both cells with a wt KSHV genome or Δ57 KSHV genome in latent infection were capable of forming SG under ARS treatment, but only the cells with a Δ57 genome in viral lytic infection did so, while the cells with a wt KSHV genome did not (Sharma et al., 2017). These data suggested that KSHV lytic protein ORF57 was most likely required for the observed suppression of SG formation.

Kaposi’s sarcoma-associated herpesvirus ORF57 is an RBP with an intrinsically disordered, non-structural N-terminal domain and a structured α-helix-rich C-terminal domain (Majerciak et al., 2015; Majerciak and Zheng, 2015; Yuan et al., 2018). Early studies from our group and others demonstrated that ORF57 functions at the post-transcriptional level for viral gene expression by enhancement of RNA stability, splicing, and translation mostly through its N-terminal domain interaction with RNAs and various RBPs (Majerciak and Zheng, 2015; Vogt and Bohne, 2016). As described above, ORF57 also inhibits PB formation during KSHV lytic infection (Sharma et al., 2019). The independent ability of ORF57 to inhibit SG formation under ARS or poly I:C treatment was confirmed in HeLa and HEK293T cells with ectopic ORF57 in the absence of other KSHV lytic proteins (Figure 4C), but not an N-terminal dysfunctional ORF57 mutant (Sharma et al., 2017). Interestingly, by using HeLa cells with ectopic expression of other individual viral proteins, we discovered that KSHV ORF37 (vSOX) bearing intrinsic endoribonuclease activity also showed the activity to inhibit ARS-induced SG formation, but KSHV RTA, ORF45, ORF59, and LANA did not display such function (Sharma et al., 2017). Homologous protein HSV-1 ICP27, but surprisingly not EBV EB2, resembles KSHV ORF57 in the ability to block the PKR/eIF2α/SG pathway (Sharma et al., 2017).

Mechanistically, ORF57 functioning as a dimer (Majerciak et al., 2015; Yuan et al., 2018) was found to directly interact with PACT, PKR, PABPC1, and eIF4E independent of RNA (Massimelli et al., 2011, 2013; Sharma et al., 2017) but has no interaction with TIA-1, G3BP1, eIF4G, or eIF2α (Sharma et al., 2017). ORF57 interacts with PACT and PKR *via* its N-terminal domain interacting with the dsRNA-binding domain of PACT or PKR (Figure 3) and blocks PACT interaction with PKR, PKR activation, and kinase activity to phosphorylate eIF2α (Sharma et al., 2017; Figure 4D). This mechanistic function of ORF57 resembles the TAR RNA-binding protein (TRBP), which inhibits PKR activity *via* the interaction with PKR (Daher et al., 2009; Daniels and Gatignol, 2012), but differs from poliovirus 3C protease, which cleaves Ras-GAP SH3 domain–binding proteins (G3BP) (White et al., 2007), and Semliki Forest virus (SFV) nsP3 and HSV type 2 (HSV-2) ICP8, which suppress SG formation by their FGDF motifs interacting with G3BP (Panas et al., 2015). KSHV ORF57 does not have such an FGDF motif and does not interact with G3BP1. Moreover, ORF57 does not inhibit heat-induced phosphorylation of elF2α and SG formation, as it is independent of the PKR pathway (Sharma et al., 2017).

Most importantly, we demonstrated that KSHV ORF57 blocking PKR activation and SG formation is one of the ORF57 functions that promote KSHV gene expression and virus production, as seen in many other virus infections. The outcome of SG formation is to trigger the host cell antiviral response and inhibit virus production (Onomoto et al., 2014). By examining KSHV virion production in iSLK-BAC16 cells bearing a KSHV genome (Brulois et al., 2012) after treatment with a PKR-specific siRNA, we found that efficient knockdown of PKR expression from iSLK-BAC16 cells led to ∼78-fold increase of KSHV virion production over the cells with the normal level of PKR expression (Sharma et al., 2017). Consistently, a recent report also revealed that KSHV infection could result in downregulation of PKR (Gabaev et al., 2020).

## Future Perspectives

Finding that KSHV lytic infection prevents the formation of RNA granules by expressing viral ORF57 represents a new dimension in understanding how KSHV evades host innate immunity for efficient viral gene expression and eventually virus production. We have learned over the years that host humoral immunity and cell-mediated immunity are two types of specific immune responses to various infections by pathogens. To date, the host innate immune system has been recognized as an equally important mechanism of intrinsic defense against invading viruses in the infected cells. This innate immune system *via* pattern-recognition receptors, including TLRs, RLRs, ALRs, NLRs, CLRs, and intracellular DNA sensors such as cGAS and IFI16 (Kawasaki and Kawai, 2014; Lester and Li, 2014; Chan and Gack, 2016; Bermejo-Jambrina et al., 2018), detects viral components such as viral genomic DNA, ssRNA, dsRNA, RNA with 5′ triphosphate ends, and viral proteins and initiates intracellular antiviral responses. KSHV evades the host immune system in multiple ways by encoding various viral proteins (Lee et al., 2015, 2016). Some of them are homologous to cellular proteins and interfere with both innate and adaptive immune responses. Immune evasion of the IFN pathway and the TLR pathway by KSHV vIRFs and other host mimics is one of the important strategies for KSHV to escape from the host innate immune response (Lee et al., 2015). KSHV vIRF-1, -2, or -3 blocks TLR3-mediated activation of IFN-responsive promoter activity, and vIRF-1 decreases phosphorylation and nuclear translocation of IRF-3 in response to TLR3 activation (Jacobs et al., 2013). We found that ORF57 also blocks poly I:C-induced phosphorylation of TLR3 (Sharma et al., 2017).

Currently, host cell RNA granules have been recognized as functional components of intrinsic defense against invading viruses in the infected cells. However, almost all viruses have evolutionarily developed mechanisms to inhibit the formation of RNA granules for their replication and multiplication in the infected cells (Zhang et al., 2019). In this regard, KSHV ORF57 functioning as a dimer (Majerciak et al., 2015; Yuan et al., 2018) has been demonstrated to interact with two major components, Ago2 and GW182 of PB and PACT and PKR of SG (Figure 3), to suppress the formation of PB and SG (Sharma et al., 2017, 2019; Zhang et al., 2019), respectively, leading to enhanced KSHV lytic infection and virus production by maintaining cellular translational machineries to function. Considering these unexpected findings, it would be important for us to address the following questions in future studies: (1) Can ORF57 function(s) that inhibit RNA granules be targeted for anti-KSHV therapeutics? (2) Can KSHV inhibition of PB through ORF57 be further explored to understand the mechanisms that regulate PB formation in mammalian cells? (3) Can identification of potential factors be associated with PKR activation during KSHV infection? (4) Can KSHV viral RNA species be determined to preferentially travel through these RNA granules? (5) Because the N-terminal half of KSHV ORF57 is intrinsically disordered like many other viral proteins (Majerciak and Zheng, 2015; Majerciak et al., 2015; Sharma et al., 2021), can the role of the intrinsic disordered region of KSHV ORF57 be ascertained in its inhibitory function toward RNA granules? (6) LSm14 (RAP55), a DDX6 interactor (Brandmann et al., 2018), is a common protein factor for assembly of both PB and SG and has the binding activity to viral RNA and DNA to mediate IRF3 activation and IFN-β induction in the early phase of virus infection (Li et al., 2012). Whether lytic KSHV infection affects LSm14 expression or its interaction with DDX6 remains to be investigated. (7) Since EBV EB2 is incapable of blocking assembly of both PB and SG, what is the distinct feature of EBV EB2 from its homologous proteins KSHV ORF57 and HSV-1 ICP27 in inhibition of PB and SG assembly? Taken together, KSHV regulation of RNA granule assembly represents an exciting area for further investigation.

## Author Contributions

NS and ZMZ drafted the review and approved the final manuscript. Both authors contributed to the article and approved the submitted version.

## Conflict of Interest

The authors declare that the research was conducted in the absence of any commercial or financial relationships that could be construed as a potential conflict of interest.

## Publisher’s Note

All claims expressed in this article are solely those of the authors and do not necessarily represent those of their affiliated organizations, or those of the publisher, the editors and the reviewers. Any product that may be evaluated in this article, or claim that may be made by its manufacturer, is not guaranteed or endorsed by the publisher.

## Footnotes

1
http://rnagranuledb.lunenfeld.ca/
